# Supplementary material for: Biophysical analysis of drug efficacy on C. elegans models for neurodegenerative and neuromuscular diseases
Source: PLoS One. 2021 Jun 11;16(6):e0246496. doi: 10.1371/journal.pone.0246496 (PMC8195402; doi:10.1371/journal.pone.0246496)
Supplement: S1 Fig — (A) Optical image showing two developmental stages of worms (L4 and young adult) thrashing on the PDMS-based micropillars. The deflection of the micropillars was used to quantify the thrashing force exerted by the worm. (B) Table showing geometric parameters for force assay chip and diameters of worms used in this study. (DOCX) [file pone.0246496.s001.docx]

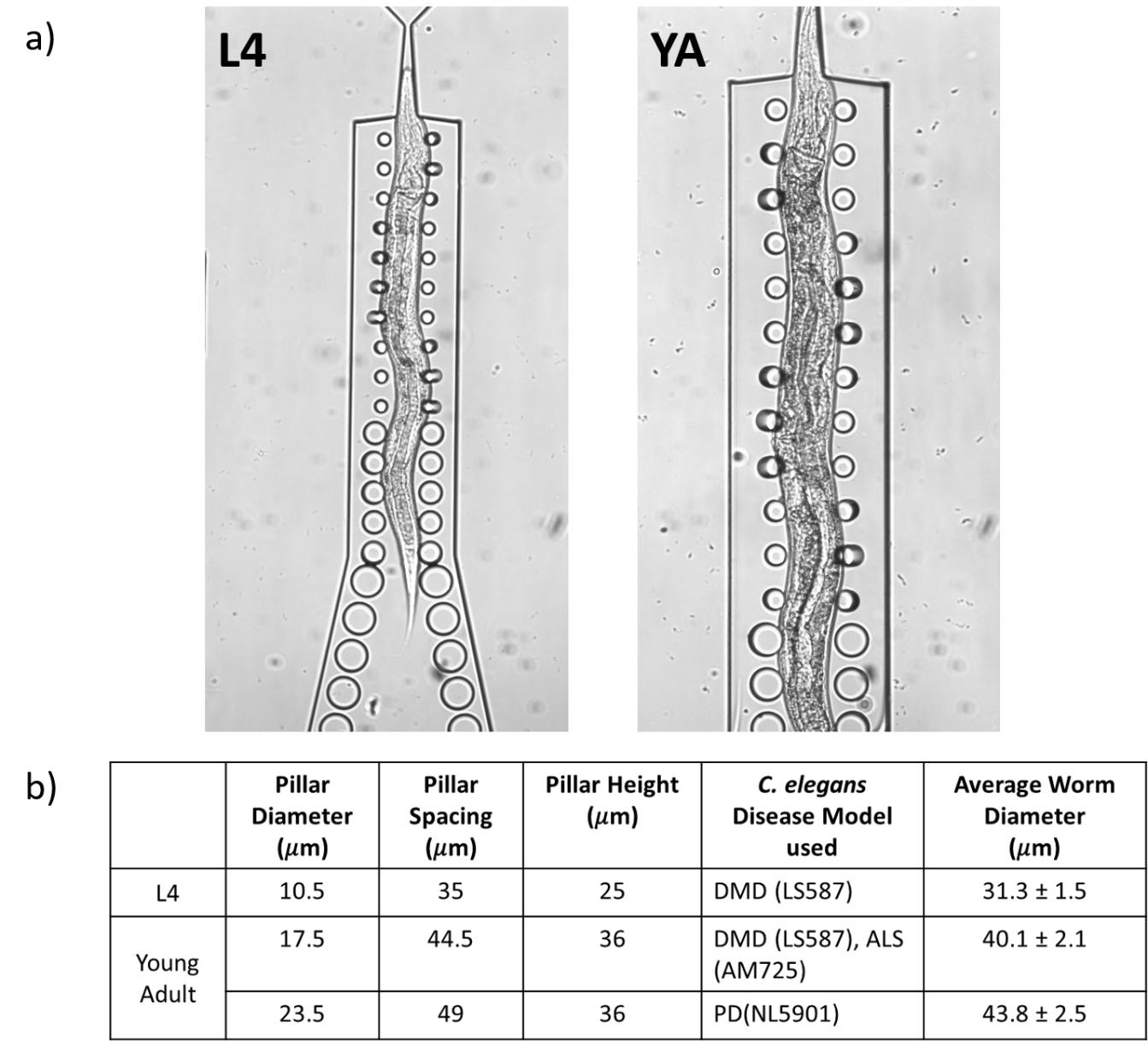


**S1 Fig: The microfluidic chip for quantifying thrashing force exerted by C. elegans**. (A) Optical image showing two developmental stages of worms (L4 and young adult) thrashing on the PDMS-based micropillars. The deflection of the micropillars was used to quantify the thrashing force exerted by the worm. (B) Table showing geometric parameters for force assay chip and diameters of worms used in this study.
