# Supplementary material for: Biophysical analysis of drug efficacy on C. elegans models for neurodegenerative and neuromuscular diseases
Source: PLoS One. 2021 Jun 11;16(6):e0246496. doi: 10.1371/journal.pone.0246496 (PMC8195402; doi:10.1371/journal.pone.0246496)
Supplement: S1 Table — (DOCX) [file pone.0246496.s002.docx]

**S1 Table: Summary of worm culture and drug treatments.**

Strain: DMD (LS587)

| Strain |  |  |  |  |
| --- | --- | --- | --- | --- |
| Drug | Dosage | Drug intake | Culture duration and stage (L4, YA) at 15°C | Solvent |
| Prednisone | 0.37mM | Mixed with NGM | -Grown from L1-L4 for ~3 days and 17 hours.  - Grown from L1-YA for ~4 days and 17 hours. | 0.062% DMSO |
| Melatonin | 1 mM |  |  | NA |

Strain: ALS (AM725)

| Drug | Dosage | Drug intake | Culture duration and stage (YA) at 20°C | Solvent |
| --- | --- | --- | --- | --- |
| Doxycycline | 10.5 μM | Mixed with NGM | -Control for ~2 days and 4 hours.  -Worms treated with 10.5 μM doxycycline for ~2 days and 9 hours.  - Worms treated with 32 μM doxycycline for ~2 days and 13 hours. | NA |
|  | 32 μM |  |  |  |
| Riluzole | 30 μM | Maintained at 20 °C until L4 stage on NGM then moved to S basal medium for drug treatment until YA | -Control incubated on NGM for ~1 day and 21 hours, while drug treated conditions incubated for 2 longer hours until L4 stage.  - Then shifted to S basal medium both control and 30 μM Riluzole were incubated for one day at 100 rpm in 96 well plate. While 100 μM Riluzole treated worms incubated for ~3 hours longer until YA stage. | 0.5% DMSO |
|  | 100 μM |  |  |  |

Strain PD (NL5901)

| Drug | Dosage | Drug intake | Culture duration and stage (YA) at 20°C | Solvent |
| --- | --- | --- | --- | --- |
| Levodopa | 0.7 mM | Mixed with NGM | Grown for ~2 days and 5 hours. | 0.5% DMSO |
|  | 2 mM |  |  |  |
| pramipexole | 2.5 mM | Drug prepared in M9 buffer and spread on top of NGM |  | M9 buffer |
|  | 5 mM. |  |  |  |
