# Supplementary material for: Biophysical analysis of drug efficacy on C. elegans models for neurodegenerative and neuromuscular diseases
Source: PLoS One. 2021 Jun 11;16(6):e0246496. doi: 10.1371/journal.pone.0246496 (PMC8195402; doi:10.1371/journal.pone.0246496)
Supplement: S2 Table — (DOCX) [file pone.0246496.s003.docx]

**S2 Table: Summary of significance test of SOD1 protein aggregate quantification before and after drug treatment.**

| **SOD1 Aggregate Parameter** | **Riluzole** | | **Doxycycline** | |
| --- | --- | --- | --- | --- |
|  | **30 μm** | **100 μm** | **10.5 μm** | **32 μm** |
| Average Size | Significant difference | Significant difference | No significant difference | Significant difference |
| Average Count | No significant difference | Significant difference | Significant difference | Significant difference |
| Average Area | Significant difference | Significant difference | No significant difference | Significant difference |
